# Utilization, receptivity and reactivity to Interactive Voice Response daily monitoring in risky drinking smokers who are motivated to quit

**DOI:** 10.18332/tid/84972

**Published:** 2018-05-30

**Authors:** Amy M. Cohn, Hoda Elmasry, Sarah J. Ehlke

**Affiliations:** 1 Battelle Memorial Institute, Arlington, United States; 2 Hoda Elmasry was at the Schroeder Institute for Tobacco Research and Policies Studies, Washington, United States at the time of data analysis and when the manuscript was submitted; 3 Old Dominion University, Norfolk, United States

**Keywords:** interactive voice response, drinking, smoking, reactivity, compliance, behavior change

## Abstract

**INTRODUCTION:**

Interactive Voice Response (IVR) technology has become an increasingly popular and valid method for collecting Ecological Momentary Assessment (EMA) data on a variety of health-risk behaviors, including daily alcohol use and cigarette smoking, and for stimulating behavior change. However, very little research has evaluated the parameters of IVR compliance and reactivity in respondents who may have greater problem severity than samples previously examined in published IVR studies. This study examined the prevalence and correlates of use, receptivity and reactivity to IVR monitoring in 77 untreated risky drinking smokers who were motivated to quit within the next 6 months.

**METHODS:**

Respondents completed twice daily IVR assessments for 28 days and were re-assessed immediately after IVR to measure receptivity and reactivity to daily monitoring and six months post-baseline.

**RESULTS:**

Mean compliance rate was 70.6%, with a morning rate of 72.4% and an evening compliance rate of 68.9% out of all possible surveys. IVR assessments of drinking and smoking were significantly associated with baseline paper-pencil reports of the same. African-American participants and those who reported more daily stressful events were more compliant. Between the baseline session and the 6-month follow-up, 68% of the sample reported engaging in some form of smoking behavior change (50% reduction in CPD, a quit attempt, past-month continuous abstinence). Nearly 80% reported increased awareness of their behavior due to the IVR and 40% reported intentional behavior change from IVR monitoring. The odds of making a quit attempt at the 6-month follow-up were significantly higher among respondents who reported making purposeful changes to their smoking as a result of IVR monitoring (AOR=3.25, p<0.05).

**CONCLUSIONS:**

Reactivity was associated with behavior change outcomes. IVR may be a useful tool for motivating behavior change in smokers with alcohol-use problems.

## INTRODUCTION

Interactive Voice Response (IVR) technology has become an increasingly popular and valid method for collecting Ecological Momentary Assessment (EMA) data on a variety of health-risk behaviors^1-3^, including daily alcohol use and cigarette smoking^4-6^. IVR data are recorded by having participants push the buttons on the keypad of their telephone to answer a set of pre-recorded survey questions. IVR offers several advantages over paper-and-pencil data collection methods^7^. It provides respondents with a level of privacy in reporting sensitive behavior; allows a shorter recall period that improves confidence of the causal sequences among factors^8^; and improves measurement of moderators and mediators of behavior by immediately time-stamping and recording data.

IVR offers several advantages over other forms of EMA, like web-based, email, text messaging or smartphone app data collection^7,9^. Other forms of EMA have a higher potential for breach of confidentiality than IVR. Data transmitted via email are not always secure because one cannot guarantee that data travel directly from point to point^10^. Further, communicating through a mobile device, such as with an app, can leave user’s personal information vulnerable to attack by viruses and malware. Additional protection must be taken to secure transmission of data sent via mobile phone or email, which may be costly or require computer programming knowledge. Similarly, text-messaging data goes through the cellular provider before it is sent to the device at the research site and often contains at least one form of identifiable information (name, phone number) and one form of private or confidential research information^10^. With IVR, however, data are recorded and stored on a password protected database and accessed only by approved study personnel. Older individuals may be less familiar with text messaging or how to use an app, making use of these EMA tools more burdensome for certain age groups. Furthermore, IVR can be deployed with any touchtone phone, thus a smartphone is not required to use IVR. Studies using more advanced EMA technologies may screen-out individuals who do not own smartphones, thereby reducing the generalizability of study findings.

The potential for missing data and lack of compliance with IVR monitoring are two limitations that can reduce statistical power and affect estimation bias^11,12^. Compliance to IVR protocols is important as it has been shown to positively affect treatment outcomes and improve treatment and medication adherence for a variety of health behaviors^13-15^. For studies with drinkers or smokers, compliance rates range significantly from 46% to over 90%^11,15-18^. Compliance may be related to the timing of assessments, the amount of time between assessments, the number of assessments per day, and the type of behaviors being assessed. Further, some respondents may have limited time on their cell phones to complete phone calls, others travel due to work or have varying access to a phone, others may be highly motivated to complete the surveys for the financial incentives, as well as for the personal growth. For alcohol use specifically, answers may be influenced by degree of intoxication at the time of the call. Assessment reactivity, or the prospect that respondents may change their behavior as a result of daily monitoring, may also result from IVR monitoring^19^, although results have been mixed. Several studies of daily diary reporting indicate that reactivity is unlikely, or if it does exist, does not significantly impact measurement validity^7,20^. However, a separate line of research suggests that IVR monitoring may be an effective tool for behavior change^13,14,21,22^.

Babor and Del Boca^23^ proposed a theoretical model that identifies the cognitive, social and psychological factors that may affect participant self-reports during the question-and-answer process, which could extend to IVR self-reporting of alcohol and smoking. Key parameters within this framework are respondent characteristics and motivational processes that may affect the use of and compliance with assessment instruments. Respondent characteristics refer to enduring qualities of the individual who is completing the assessment, such as personality traits or demographic factors. Motivational characteristics are those factors that could affect the extent to which a respondent conforms to and complies with the assessment instructions, including substance use severity and desire to change behavior.

Individuals with co-occurring substance use disorders, like smoking and alcohol use, may experience unique barriers in complying with the IVR, and may react to the IVR differently than those without comorbidity. First, individuals with more severe symptomatology may have difficulty in maintaining motivation to continuously engage with the IVR system. Three prior studies with samples of drinkers showed that IVR compliance was inversely related to alcohol problem severity^24,25^. It is possible that respondents with concurrent nicotine dependence and alcohol-use problems may have lower IVR compliance than those reported in the current literature, which has focused on either smokers or drinkers, as separate groups^5,12,24,25^; none has focused on smokers who are also risky drinkers. Second, concerns have been raised about whether cognitive, emotional or physical impairments associated with substance-use behavior may interfere with one’s ability to complete IVR surveys^26^. Numerous studies in the alcohol-treatment literature show a strong correlation between chronic and heavy drinking with neuropsychological impairment, including problems with attention and working memory^27^. Smoking has also been cited as a risk factor for dementia and decline in cognitive abilities^28^, factors that could interfere with reporting behavior, particularly in smokers who consume a pack or more of cigarettes a day. Third, individuals with more severe problems may also have limited funds to pay for the minutes accrued by each IVR survey, thus reducing their overall adherence to the survey schedule. One recent study showed that drinkers with greater financial stability were more compliant with the IVR system^24^, suggesting that the financial status of the respondent may be an important indicator of IVR utilization. Fourth, individuals who smoke and drink may also experience a wide array of psychosocial stressors (occupational distress, health or financial problems), which could interfere with motivation, with their ability to answer calls promptly, or to complete surveys in the allotted time frame. Individuals with more severe substance-use problems may want to minimize the extent of their use behavior and could be reluctant to engage in self-monitoring protocols if they are less motivated to change. They could be concerned that daily tracking would reveal daily, and perhaps harmful patterns of use they may wish to ignore or to overlook. Finally, those who are highly motivated to change their substance use may find frequent or daily monitoring particularly useful, as it may increase awareness of their use patterns and provide insight into ways to alter these patterns.

More research is needed to evaluate the parameters of IVR compliance and reactivity in respondents who may have greater problem severity than samples previously examined in published IVR studies. No study, to our knowledge, has examined factors associated with IVR compliance in a comorbid group of smokers who drink at risky levels. The lack of published literature in this area is noteworthy, because the co-use of alcohol and tobacco is more prevalent among US adults than use of either substance alone^29^. To fill this knowledge gap, the first aim of this study was to examine the prevalence and correlates of IVR compliance in a sample of smokers with comorbid alcohol use disorders (AUDs) and alcohol-related problems, specifically to determine if IVR compliance varies with substance-use severity. Based on findings from prior studies, we hypothesized that age, income and motivation to change drinking and smoking would be positively associated with compliance; while greater alcohol consumption, nicotine dependence severity, negative daily life events, and the desire to use cigarettes or alcohol to cope with problems would be inversely associated with IVR compliance. Given that IVR monitoring can increase awareness of behavior and promote behavior change, the second aim was to examine the receptivity and self-reported reactivity to IVR (i.e. behavior change in response to IVR monitoring), as well as the impact of reactivity on smoking behavior change outcomes at a 6-month follow-up. While one of the pitfalls of EMA may be distortions or inaccuracies of data collection due to unintentional behavior change from self-monitoring, this may also be a strength of EMA. Individuals may be able to make changes to their behavior, or improve their motivation to change, through low-cost and broad-reaching self-monitoring protocols.

## METHODS

### Participants

Data were collected in a large Northeastern city in the United States between 2013 and 2015 by trained Masters’ level study personnel. Participants were 84 risky drinking smokers recruited via print and web-based advertisements that asked for ‘smokers who are regular drinkers’. Inclusion criteria were: 1) age 18-65 years, 2) smoked > 10 cigarettes/day, 3) consumed alcohol at NIAAA-defined risky drinking levels [> 2 drinks/day and 14 drinks/week for men; >1 drink/day and >7 drinks/week for women]^30^, and 4) reported a desire to quit smoking within the next 6 months. Exclusion criteria were: 1) severe psychiatric disturbance, 2) potential for significant alcohol withdrawal, 3) use of cocaine, heroin or crack, 6 out of 7 days a week in the past month^a^, 4) or pregnant or planning to become pregnant. Using criteria that were based on the clinical practice guidelines for treating tobacco dependence^31^, desire to quit smoking in the next 6 months was measured on a five-point scale, with response options: 1) *yes, definitely*, 2) *yes, probably*, 3) *unsure*, 4) *no*, *I would rather not*, or 5) *no, definitely not*. Individuals who answered *no, I would rather not,* or *no, definitely not* were not eligible. Inclusion/exclusion criteria were assessed via self-report and semi-structured interview. This study was approved by the Schulman IRB, protocol # 201304003. All data were collected in accordance with the Declaration of Helsinki.

### Baseline measures

Measures listed below are standard instruments commonly used in smoking and/or alcohol research studies and are psychometrically valid and reliable. After providing informed consent, demographic information, including gender, race, ethnicity, income and employment were collected. Readiness to quit smoking was assessed using the Contemplation Ladder (CL), a self-report instrument that measures readiness to quit smoking on a 10-point Likert-type scale (‘no thoughts of quitting’ to ‘taking action to quit’)^32^. The CL has shown good convergent validity with other measures of motivation to change, and predicts longer term readiness to quit smoking in samples of adults^32-34^. The Stages of Change and Treatment Readiness Scale (SOCRATES)^35^ is a 19-item self-report instrument that was used to assess motivation to change drinking behavior (1 - *strongly disagree* to 5 -*strongly agree*) and contains three subscales: *Ambivalence, Recognition,* and *Taking Steps*. The Smoking Temptations Inventory (STI)^36^ is a 9-item validated self-report measure that was used to assess temptation to smoke in nine high-risk smoking situations (1 - *not at all tempted* to 5 - *extremely tempted*). The STI has demonstrated good reliability and internal consistency in samples of adult smokers^36^, with a total score reflecting overall temptation to smoke. The SOCRATES demonstrates excellent test-retest reliability in drinkers and good convergent validity with measures of longer term drinking^35^. Nicotine dependence was measured using the 6-item self-report Fagerström Test for Nicotine Dependence (FTND)^37^, which has demonstrated high reliability and validity, and good internal consistency in samples of daily smokers. The Structured Clinical Interview for DSM-IV (SCID)^38^ is a semi-structured interview that was used to assess lifetime and current alcohol use disorder (AUD), as well as number of lifetime AUD symptoms. The SCID was administered by Master’s level trained interviewers and all SCIDs were reviewed by the PI of the study. The Time Line Follow Back interview (TLFB )^39^ is an interviewer administered questionnaire that was used to assess day-level drinking and smoking behavior within the 90 days prior to the baseline assessment. The TLFB yielded the following indices that were used in the current analyses: mean drinks per drinking day (MDDD), proportion heavy drinking days (6+ drinks/day, PHDD), and cigarettes per smoking day (CPD) within the 90 days prior to the baseline session. Our threshold for PHDD aligned with criteria from the Alcohol Use Disorder Identification Test^40^. The TLFB has shown high test-retest reliability, and strong correlations between participant and collateral reports of drinking and smoking^39,41^.

### IVR assessments

At the baseline session, participants completed a 20-minute IVR training, wherein they were taught to record drinking data (in standard drinks) and completed a practice IVR survey from their telephone. Participants were instructed to record their responses to pre-recorded survey questions by pushing buttons on the keypad of their telephone.

Participants recorded daily factors in the following areas: 1) quantity and frequency of beer, wine and liquor consumed in standard drink conversions since the previous assessment; 2) cigarettes consumed since the previous assessment; 3) degree of temptation to smoke in nine high-risk situations since the previous assessment (items adapted from the STI; 1 - *not at all tempted* to 5 -*extremely tempted*)^36^; 4) smoking cigarettes to cope with any negative daily events since the previous assessment (*yes/no*); 5) drinking alcohol to cope with any negative daily events since the previous assessment (*yes/no*); and 6) the occurrence of specific life events since the previous assessment (argument with a friend or family member, a financial problem, a work-related problem, and a problem related to their health or well-being issue). Other factors known to correlate with drinking and smoking, including mood and craving, were also included in the daily assessments but are not reported in this paper. IVR assessment items were selected to parallel the constructs measured at baseline (e.g. self-efficacy) and have established psychometric properties^2,42,43^.

For 28 days following the baseline, participants recorded daily alcohol use, smoking, smoking temptation and smoking-related risk factors. Prompts (e.g. calls to their telephone) were programmed electronically to occur twice a day at random times within each of two 4-hour periods, one corresponding to the morning and one the evening. The hour of administration for the morning and evening surveys differed for each participant, as prompts were programmed to coincide with the respondents’ sleep-wake cycle (i.e. the usual times they wake up and go to sleep), which was collected at the baseline survey. The IVR system was configured to call (prompt) the participant’s cell phone and was enabled so that participants could directly access the survey after they received the call by pressing ‘1’ on their phone keypad. Prompting lasted for 10 seconds, and a participant had 2 minutes to respond. If the prompt was missed, the IVR system cued two additional prompts, each 5 minutes apart. Because data were time-stamped, we recorded whether surveys were completed outside of the scheduled sleep-wake cycle. The IVR system was set up so that no random prompts were issued within 2 hours of each other, and separate morning and evening interviews were programmed to facilitate different questions at each time period. IVR interviews were date- and time-stamped and recorded immediately. Several system features were used to promote adherence, including clear prompts, minimal skip outs, and ability to return to questions.

To enhance IVR compliance, participants received $15 each week they participated in the IVR phase (total of $60). Additional bonus incentives were provided at $0.50 for each completed call; as well as $2 per week for completing prompts 6 out of 7 days, or $5 per week for completing prompts for all 7 days. Thus, participants were eligible to receive up to $108 if they completed all IVR interviews.

### Post-IVR survey

At the end of IVR monitoring (1-month post-baseline), participants completed a questionnaire to measure receptivity and reactivity to IVR. The post-IVR survey was developed specifically for this research study, in collaboration with several experts in the field of EMA data collection. To measure receptivity, participants were asked: ‘Did you feel the monitoring schedule was burdensome or took too much time?’, and ‘Were the questions easy to understand?’. All items used the same 5-point scale (0 - *Not at all*, 4 - *Extremely*). To measure reactivity, participants were asked: ‘To what extent did you feel that the daily phone calls may have caused you to be more aware of your behavior?’ (0 - *Not at all*, 4 - *Extremely*), and ‘Did you begin to notice any behaviors more than before, and if so which ones? (select all that apply)’, with response options including smoking and alcohol use (*yes/no*). Participants were also asked: ‘Did you find that you purposely started to make changes to your behavior because of the daily monitoring?’ (0 - *Not at all*, 4 - *Extremely*) and ‘which behaviors did you purposely make changes to? (select all that apply)’, with response options for smoking and alcohol use (*yes/no*).

In total, 85% of the sample (n=72) completed the post-IVR survey. Non-completers had a higher income [F(1,74)=4.79, p<0.05], and were more likely to be male (χ^2^=4.99, p< 0.05).

### Six-month follow-up

A follow-up assessment of alcohol and smoking behavior change was completed 6-months post-baseline and included re-administration of the TLFB to obtain measurements of smoking and alcohol use behavior, as well as measurements of 7-day and 30-day point prevalence abstinence from smoking. Those who reported not smoking for at least 24 hours were considered having made a quit attempt.

In total, 84% completed the 6-month follow-up (n=71). Those who completed the 6-month survey were more likely to be African-American (χ^2^=7.39, p< 0.05).

### Analytic plan

We assessed two compliance rates, one for percentage of surveys answered over the course of the study, and the other for percentage of survey questions answered over the course of the study. The overall compliance rate for surveys was based on the number of surveys in which at least one question in that survey was answered, divided by the total number of surveys possible. The denominator was the total number of morning and evening surveys multiplied by the total number of participants ([28 morning surveys + 28 evening surveys]x77 participants = 4312, the total number of surveys in the study). Given the study design (separate morning and evening surveys), overall compliance rate was also stratified by time of day, yielding compliance rates overall, as well as in the morning and in the evening. Weekend vs weekday compliance rates, as well as average survey response times were also examined. Question-level compliance rate was the number of all questions answered across all study days and participants, divided by the total number of possible questions that could be answered across all study days and participants. Because there were 47 total possible questions in the morning survey, 40 total possible questions in the evening survey, 28 study days and 77 respondents, the denominator for this analysis was equal to 187572 ([47 + 40]x28x77).

Descriptive statistics examined differences in compliance rates and missed surveys overall, based on daytime-evening and weekday-weekend reports. Correlation coefficients were then obtained to examine the association between IVR-assessed measurements of smoking, drinking and smoking temptation with corresponding baseline paper-pencil reports. Next, linear regression models were performed to examine the association of demographic, tobacco and alcohol use factors, motivation to change, and daily life events (i.e. argument with a family or friend, financial problems, health problems) with compliance rates, to determine if compliance rates varied as a function of greater problem severity, motivation to quit smoking, and stressful life events.

Prevalence, correlates and predictive validity of IVR receptivity and reactivity were then examined. Regression models (binary logistic or linear regression, depending on the outcome) examined the predictive utility of IVR reactivity on change in alcohol and smoking behavior from baseline to 6-months. The independent variables for these models were increased awareness of smoking (or drinking) and purposeful changes to smoking (or drinking) resulting from IVR self-monitoring. Change in smoking behavior from baseline to 6-months was examined in several different ways. First, using linear regression analysis, we examined changes in cigarettes/day and nicotine dependence severity (as separate outcomes), controlling for baseline reports of the outcome, motivation to quit smoking, and IVR compliance (% of surveys completed). Second, a variable was created to examine overall smoking behavior change, based on reports of at least one of the following (dichotomous, yes/no): 1) a 50% reduction in cigarettes smoked per day from baseline to the 6-month follow-up; 2) cessation attempt lasting > 1 day and < 7 days; or 3) past-month continuous abstinence from smoking. This broad definition of smoking behavior change was chosen to include a variety of different change outcomes, similar to another study^44^. Each outcome was also examined in separate models, with the exception of past-month continuous abstinence, due to small cell size (n=1). Because this outcome was dichotomous, bivariate logistic regression analyses were employed, using the independent variables capturing increased awareness of smoking and purposeful changes to smoking.

For changes in alcohol use behavior, linear regression analyses were used to examine changes in percentage heavy drinking days and mean drinks per drinking days from baseline to 6-months, controlling for baseline reports of the outcome, motivation to quit smoking, and IVR compliance.

## RESULTS

### Participant characteristics

Table 1 shows participant characteristics of the 77 respondents who took at least one IVR survey. Participants were middle aged adults (M=45.8, SD=10.8), unmarried (84%), African-American (86%), and male (52%). Nearly all participants had either a lifetime AUD (93.5%) or a past-month AUD (64.9%). Participants smoked 13.8 cigarettes/day (SD=7.4), reported a moderate degree of nicotine dependence (M=6.4), and consumed 7.4 drinks (SD=5.6) per drinking day at baseline. Even though they were heavy drinkers, most (90%) indicated little recognition of the need to make changes to their drinking and 89.6% was not actively taking steps to change their drinking.

**Table 1:** Participant characteristics n = 77 respondents

*Demographic factors*		
	Mean	SD
Age	45.6	10.8
	N	%
Gender		
Female	37	48.1
Male	40	52.0
Race		
White	7	9.1
African American	66	85.7
Other	4	5.2
Employed	39	50.6
Not employed	38	49.4

***Baseline alcohol and tobacco use factors***		

	Mean	SD
Temptation to smoke	34.18	7.48
Mean drinks per drinking day	7.43	5.63
Proportion heavy drinking days (6+ drinks/day)	31%	34%
Cigarettes per day	13.83	7.42
Nicotine dependence	4.16	2.24
Readiness to quit smoking (contemplation ladder)	6.28	2.28
	N	%
Motivation to change drinking (SOCRATES)		
Actively taking steps to change drinking		
High	8	10.4
Low	69	89.6
Ambivalence about changing drinking		
High	33	42.9
Low	44	57.1
Recognition of need to change drinking		
High	7	9.1
Low	70	90.9
Alcohol use disorder (AUD)		
Lifetime	72	93.51
Current	50	64.9

### Compliance rates, missed reports and survey completion time

Table 2 shows overall compliance rates and missed reports by time-of-day and by weekend vs weekday. Participants completed an average of 39.56 out of 56 total possible surveys (n=3046 total surveys), yielding an average compliance rate of 70.6% (SD=29.79, range: 1.79–100), with a morning rate of 72.4% and an evening compliance rate of 68.9% out of all possible surveys that could have been completed for those periods. There were significantly higher compliance rates in the mornings compared to the evenings (t: 2.48, p<0.01) and on weekdays (71.7%) vs weekends (68.1%) (t: 2.35, p<0.05). A third of the sample (34%) completed all 28 IVR days, only 3 participants completed just one day. Initial compliance rate was 75.7% in week 1 and fell to 65.9% by week 4.

**Table 2:** Compliance rates across morning and evening, weekday and weekend, surveys out of n = 4312 total possible surveys and missed surveys

*Survey compliance (_nmax_=4312 )*		
Overall surveys	3046	70.6
Study days with either morning or evening completed surveys	1707	79.2
Morning vs Evening		
Morning	1560	72.4
Evening	1486	68.9
Weekend vs Weekday		
Weekend	840	68.1
Weekday	2206	71.7

***Participants with missing surveys (n=77 )***		

Overall	N	%
Missed 0-5 surveys	28	36.4
Missed 6-10 surveys	9	11.7
Missed 11+ surveys	40	51.9
Morning Surveys		
Missed 0-5 surveys	44	57.1
Missed 6-10 surveys	11	14.3
Missed 11+ surveys	22	28.6
Evening Surveys		
Missed 0-5 surveys	40	51.9
Missed 6-10 surveys	10	13.0
Missed 11+ surveys	27	35.1

In terms of missed reports aggregated across both morning and evening surveys, 36% missed up to five of the 56 possible surveys; 11.7% missed between 6 and 10 surveys; and half missed 11 or more surveys. The breakdown between morning and evening surveys was slightly different, with the majority missing up to 5 surveys for both morning and evening; a quarter of the sample missed 11 or more morning surveys; and over a third (35%) missed 11 or more evening surveys.

Survey completion time was 5.6 minutes (SD=2.2) and decreased significantly over the course of the study (β: -2.5, p<0.0001). One third of evening surveys (33.9%) and slightly fewer (19%) of the morning surveys were taken outside of the predefined sleep-wake cycle collected at baseline. The compliance for all survey questions taken was 67.4% (SD=41.2).

### Association between IVR and paper-pencil reports

Aggregated daily responses of smoking temptation from the IVR-administered STI were significantly and positively correlated with baseline paper-pencil administered STI scores (r=0.53, p<0.001). Aggregated daily responses of smoking (r=0.27, p=0.02) and drinking (r=0.61, p<0.001) were also significantly correlated with corresponding baseline TLFB scores. Cronbach alpha for the 9-item IVR-administered STI was high (α=0.94) for both the morning and evening assessments, which was slightly higher than the value (α=0.86) for the baseline paper-pencil administration of the STI.

### Baseline and daily correlates of IVR compliance

Table 3 shows results of bivariate linear regression models of demographic and baseline correlates of IVR compliance (continuous outcome), with variables entered in the model individually. Compliance rates among White participants (M=49.5±23.7) were significantly lower than African-American participants (M=74.2±28.7, p=0.03). No other significant baseline and demographic correlates of IVR compliance emerged^b^.

**Table 3:** Bivariate regressions of demographic and baseline correlates of IVR survey compliance

	*Survey compliance rate*		
	*β*	*SE*	*p*
**Demographic factors**			
Gender			
Female	3.28	6.83	0.63
Male (ref)			
Age	0.15	0.32	0.64
Race			
White	-24.67	11.48	0.03
African American (ref)			
Other	-24.61	14.87	0.10
Employment status			
Employed	1.56	6.83	0.82
Not employed (ref)			
**Baseline factors**			
Temptation to smoke	0.66	0.46	0.15
Mean drinks/drinking day	-0.33	0.61	0.59
Proportion heavy drinking days	-4.89	9.84	0.62
Cigarettes per day	0.48	0.46	0.30
Nicotine dependence	1.43	1.53	0.35
Readiness to quit smoking	1.27	1.43	0.37
Motivation to change drinking (SOCRATES)			
Actively taking steps to change drinking			
High	14.8	11.1	0.18
Low (ref)			
Ambivalence			
High	-0.42	6.91	0.95
Low (ref)			
Recognition of need to change drinking			
High	-19.9	11.7	0.09
Low (ref)			
Alcohol use disorder (AUD)			
Lifetime	-5.31	7.69	0.49
Current (ref)			
No	5.71	14.09	0.69

SOCRATES: Stages of Change and Readiness and Treatment Eagerness Scale

Table 4 shows results of linear regression models examining the association between aggregated daily events with overall compliance rates. We conducted these analyses because we proposed that respondents experiencing more daily hassles and stressful life events may be less compliant with the IVR system. There were significant positive associations between arguments with family or friends, financial problems, drinking and smoking to cope, with overall compliance rate (all p<0.05).

**Table 4:** Association between aggregated daily events (over 28 days) and overall compliance rate

*Daily event*	*Mean*	*SD*	*β*	*R*^2^	*p*
Argument with family	7.30	9.59	0.94	0.09	0.01
Work problem	2.84	6.87	0.75	0.03	0.13
Argument with friend	6.38	10.49	0.76	0.07	0.02
Financial problems	11.79	15.82	0.56	0.09	0.01
Health problems	5.73	11.76	0.41	0.03	0.16
Drinking to cope	10.33	14.05	0.63	0.09	0.01
Smoking to cope	15.99	17.49	0.59	0.14	0.001

Mean indicates average number of times participants reported the event occurring over the 28-day EMA monitoring period, aggregated across all respondents.

### Prevalence, correlates and predictive utility of IVR receptivity and reactivity

**Figure 1:**
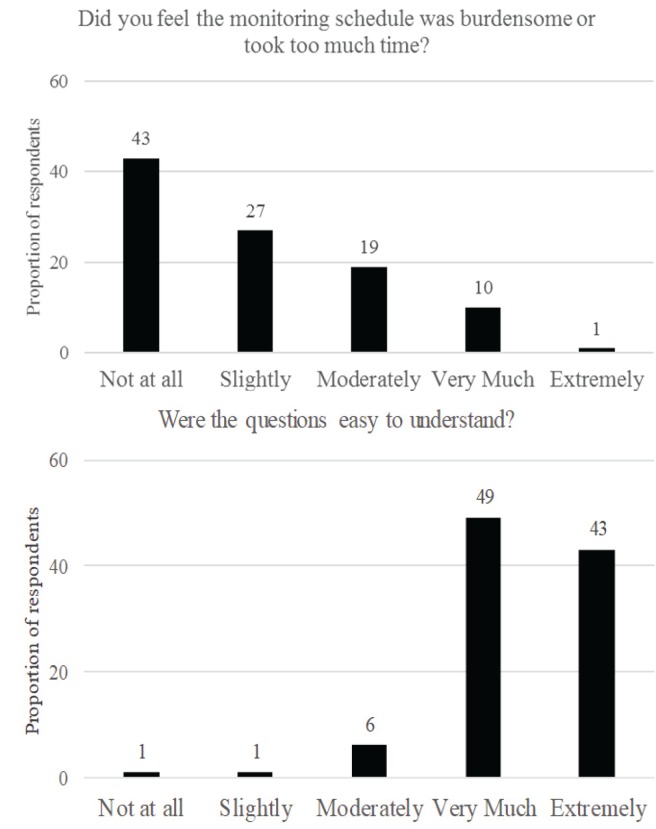
Proportion of respondents reporting that IVR monitoring was burdensome and easy to understand

Figure 1 shows the proportion of respondents who provided receptivity ratings for the IVR monitoring protocol. The majority (70%) reported that the IVR was ‘not at all’ or ‘slightly’ burdensome, and ‘very much’ or ‘extremely’ easy to understand (83%).

**Figure 2:**
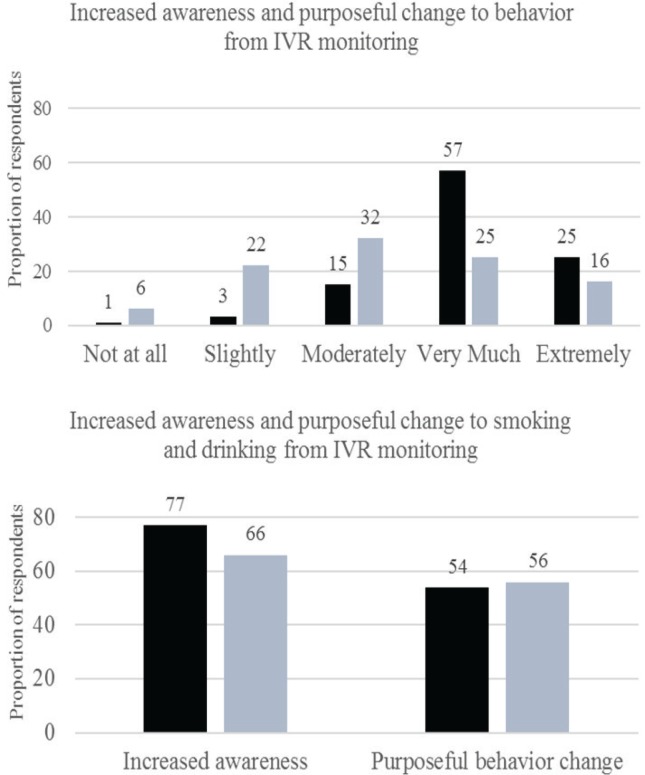
Proportion of respondents reporting increased awareness and purposeful behavior change from IVR monitoring

Figure 2 shows the proportion of respondents who reported increased awareness of their behavior due to IVR monitoring and purposeful change to their behavior from IVR. Most participants (80%) reported that IVR monitoring caused them to be ‘very much’ or ‘extremely’ more aware of their behavior in general; 77% reported greater awareness of their cigarette smoking; and 66% reported greater awareness of their drinking. In total, 56% of respondents reported making intentional changes to their drinking, and 54% reported making intentional changes to their smoking. IVR compliance (% of reports completed out of the total) was unrelated to intentional change to smoking or drinking, or to increased awareness of these behaviors.

Between the baseline session and the 6-month follow-up, 68% of the sample reported engaging in some form of smoking behavior change (50% reduction in CPD, a quit attempt, past-month continuous abstinence); with 40% making an intentional quit attempt, 35% showing a 50% reduction in cigarettes/day, and one person reporting past-month continuous abstinence. Results from adjusted logistic regression models showed that the odds of engaging in some form of smoking behavior change at the 6-month follow-up were nine times larger among respondents who reported increased awareness of their smoking compared to those who did not (AOR=9.53, p<0.01), even after controlling for baseline motivation to quit smoking and IVR compliance. The odds of making a quit attempt at the 6-month follow-up were three times greater among respondents who reported making purposeful changes to their smoking, as a result of the IVR, compared to those who did not (AOR=3.25, p<0.05). There was a trend for those who reported increased awareness of their behaviors (overall) to engage in some form of smoking behavior change (AOR=1.56, p=0.08), to make a smoking quit attempt (AOR=2.06, p=0.05), and to make reductions in their percentage of heavy drinking days (B= -0.06, p=0.10). No other significant effects of IVR reactivity were found.

## DISCUSSION

This is the first study to evaluate the use of and receptivity to daily IVR assessments in a sample of untreated risky drinking smokers who were motivated to quit smoking. Most other EMA studies have included treated samples of drinkers and/or smokers, but these do not capture the naturally occurring changes in smoking and alcohol-related factors in untreated samples without the influence of intervention. Calls were completed on nearly 80% of days, with an overall compliance rate of 70%, and individuals were more likely to comply with the survey in the mornings and on weekdays. Surveys lasted approximately 5 minutes, but survey completion time significantly decreased over time. Participants appeared to be accurately reporting their substance-use behavior over the course of 28 days, as evidenced by significant and positive associations between IVR-assessed cigarette and alcohol use, and smoking temptation with corresponding baseline paper-pencil reports. Cronbach’s alpha for the IVR-administered smoking temptation questionnaire was also high, providing further evidence that participants were not haphazardly responding to survey items. There were no obvious demographic or substance use factors, aside from race, that negatively affected compliance rates and that could have identified participants, at the outset of the study, in need of additional assistance and support utilizing the IVR system. Motivation to quit smoking could have been unrelated to IVR compliance because individuals who were in the study already had a high desire to quit smoking. Further, it is possible that the association between White race and lower compliance was a spurious effect, perhaps due to unequal numbers of White and Black respondents in the sample, as tests of mean differences can be affected by unequal cell sizes^45^. Replication of our study findings are warranted to determine whether compliance is differentially affected by race or ethnicity and whether generalizable.

Surprisingly, individuals who reported greater interpersonal and financial problems, and reported using drinking and cigarette smoking as forms of coping, were more compliant with the system. It is possible that those who experience more negative life events and who use cigarettes or alcohol to cope with these problems may have been using the IVR as a form of treatment or emotional support. This notion is consistent with other work showing that drinkers with more severe problems and consequences of use will make changes to their drinking, perhaps because they recognize the need to do so or because they are compelled to do so by family, friends or occupational circumstances^46^. The low-cost, convenience and confidentiality of IVR may make it a more acceptable method for behavior change than formal face-to-face treatment. It is also important to note that those who experienced financial problems had higher compliance rates. It is possible that because participants were compensated for their survey compliance, those with greater financial problems were more likely to be compliant in an effort to be paid more. Regardless, this finding suggests that contingency management and incentive-based interventions, which provide rewards for abstinence and behavior change, may be effective at enhancing compliance rates to a variety of treatment regimens in this group of smokers, including medication adherence, treatment session adherence, etc.

Participants overwhelmingly rated the IVR as easy to understand and to complete. This should allay some concerns that IVR surveys are burdensome and could potentially cause reporting fatigue. The majority of participants reported increased awareness of their drinking behavior and made intentional changes to this behavior as a result of IVR monitoring, but they did so regardless of the number of times they used the system. Further, the odds of making a quit attempt were significantly greater among those who reported increased awareness of their smoking behavior and intentional changes to their smoking post-IVR. This finding is somewhat inconsistent with that of Hufford et al.^20^ who showed minimal impact of EMA recording on behavior change, but their sample differed from ours in that they monitored college students who were not heavy drinkers or regular smokers, the monitoring period in their study was much shorter (2 weeks), and also our sample was motivated to make changes. While our findings suggest that IVR may be useful at increasing awareness of problematic substance-use behavior, this enhanced awareness does not appear to translate into robust behavior change, as no other indices of self-reported reactivity predicted alcohol or cigarette smoking behavior change. Further, it is important to note that respondents said that IVR helped stimulate behavior change, although there was no significant association between compliance rates and these self-reported measurements of reactivity. It is possible that respondents are unaware of why they made changes, or that even a small amount of IVR monitoring can have an impact on changing attitudes and behavior. We cannot rule out whether the link between reactivity and subsequent behavior change was due to natural shifts in behavior over time, as respondents who self-selected into the study were already highly motivated to quit smoking. It is important to note that respondents were told during informed consent that the purpose of the study was to examine associations of daily smoking and drinking patterns on subsequent cessation behavior. It is possible that this information could have affected IVR reactivity. Similar to Hufford et al.^20^, we did not have a non-EMA control group to experimentally compare change outcomes. Taken together, findings suggest that special populations are receptive to IVR monitoring, and that IVR may help motivated smokers to make positive behavior change. The extent to which daily monitoring can be supplemented with more intensive forms of treatment (psychotherapy, pharmacotherapy) to enhance motivation to change and longer term change outcomes, should be examined.

The findings of this study demonstrate that using IVR to collect daily smoking and drinking behavior in smokers with co-occurring alcohol use problems is feasible and provides psychometrically valid data. We have shown that attitudinal and behavioral change in response to IVR monitoring is possible in a sample that is not likely to seek formal treatment. This has important implications for the use of low cost, broad-reaching assessments or brief interventions with sub-groups who have substance-use comorbidities. We are unaware of any study that has directly examined changes in attitudes or behavior following IVR monitoring and the impact of such self-reported changes on subsequent natural change outcomes in a sample of risky drinking smokers who are not seeking treatment. This approach allowed us to capture the potential impact of daily self-monitoring on naturally occurring changes in smoking and alcohol use, without the influence of counseling or medication. While there was convergence across IVR-administered measures of smoking and drinking with corresponding paper-pencil reports, the strength of associations was not large^47^. This further highlights that daily assessments provide qualitatively different information than what can be measured via traditional paper-and-pencil questionnaires^8^.

There are several limitations of this study. First, retrospective reports of drinking and smoking were not collected at the end of the IVR to cross-validate behavior over the 28-day reporting period. Other studies have taken such an approach and found high levels of correspondence between IVR and retrospective measurements of smoking and drinking^5,48^. Second, most participants in the current study were African-American and findings may not be generalizable to other groups of heavy drinking smokers. Rates of current smoking are almost three and a half times higher among Black than White adults in the US^49^, making this an important target population for future research. Third, data are correlational in nature and causal conclusions cannot be made. We cannot determine whether daily stressful events predict compliance rates, or whether degree of compliance somehow affects the intensity and number of stressful events reported, perhaps through increased awareness of these events. Because we used aggregated assessments of daily stressful events, we did not control for differences across participants with respect to the number of reports they provided. Fourth, we are unable to determine whether self-monitoring had an impact on changes in attitudes and behaviors, beyond no self-monitoring. Fifth, intoxication at the time of the IVR assessment was not queried. However, daily reports with drinkers indicate that participants can reliably and accurately report on the IVR with a high degree of detail even when intoxicated^4,50,51^. Finally, several other IVR studies have used similar, if not smaller, samples when reporting compliance rates of alcohol, substance use, or other behaviors, thus allaying potential concerns about the study sample size.

## CONCLUSIONS

Our findings support the feasibility of using IVR with smokers who are heavy drinkers and show that pre-study risk factors (i.e. alcohol use problem severity, nicotine dependence) do not affect the degree to which these individuals are likely to comply with the IVR system. Further, IVR appeared to help stimulate and enhance the process of positive behavior change in a group motivated to make changes. Identifying barriers to implementing and executing IVR with high-risk populations could inform modifications to current and ongoing EMA protocols to improve efficiency and reduce participant burden. Further, isolating factors that can influence utilization and reactivity to IVR monitoring can lead to the provision of improved guidelines regarding the use of IVR as a brief screening and intervention tool.

## Footnotes

a Given human subjects concerns, we excluded participants with active suicidal or homicidal features, or whose stimulant or opiate use represented an immediate health hazard, because the proposed study design did not address or treat these symptoms and had the potential to place the participant at greater risk for medical and psychological problems relative to the benefits of participation. Because the primary aim of this study was to examine the longitudinal behavior change outcomes of untreated problem drinking smokers, those who provided evidence of these behaviors at the time of the screening interview were given referrals for treatment, but were no longer considered eligible for the study. We considered the impact of excluding these people on the overall science of the project, but felt it was more important to provide referrals for treatment than to observe these individuals potentially decline or decompensate in their mental health or substance use over the course of the study without intervening.

b Adjusted regression models were also conducted using a backward elimination stepwise procedure and a model building approach wherein variables were entered one at a time and then model fit was assessed. For backward elimination, variables were entered in the following groups: demographics, alcohol-related variables, and tobacco variables. Findings from both approaches did not yield significantly different findings from the bivariate analyses presented in the paper.
